# Exploring olive mill wastewater as a reservoir of antimicrobial and antioxidant compounds: insights from a cross-regional Moroccan study

**DOI:** 10.3389/fmicb.2026.1762240

**Published:** 2026-05-29

**Authors:** Khaoula Faiz, Chaymae Ghaffouli, Soumia Ait Assou, Adil Roukbani, Sahar Abdulaziz AlSedairy, John P. Giesy, Mohammed Benlemlih, Mourad A. M. Aboul-Soud, Bouchra Louasté

**Affiliations:** 1 Laboratory of Biotechnology, Environment, Agri-food and Health, Faculty of Sciences Dhar El Mahraz, Fez, Morocco; 2 Department of Food Sciences and Nutrition, College of Food and Agricultural Sciences, King Saud University, Riyadh, Saudi Arabia; 3 Department of Environmental Sciences, Baylor University, Waco, TX, United States; 4 Biomedical Sciences and Toxicology Centre, Western College of Veterinary Medicine, University of Saskatchewan, Saskatoon, SK, Canada; 5 Department of Integrative Biology and Center for Integrative Toxicology, Michigan State University, East Lansing, MI, United States; 6 Center of Excellence in Biotechnology Research (CEBR), College of Applied Medical Sciences, King Saud University, Riyadh, Saudi Arabia

**Keywords:** HPLC-DAD profiling, hydroxytyrosol, minimum inhibitory concentration, olive mill wastewater, radical scavenging activity, waste valorization

## Abstract

Olive oil producing countries, including Morocco, generate large volumes of olive mill wastewater (OMW), which is characterized by high organic and phenolic loads, leading to serious environmental concerns. The present study aimed to evaluate this by-product by investigating its antioxidant and antimicrobial potential. Twelve OMW samples collected from five Moroccan regions were subjected to this study. Polyphenol extraction yielded total phenolic contents ranging from 183.49 ± 2.18 to 736.56 ± 2.94 mg GAE/g extract and total flavonoid contents between 43.22 ± 0.42 and 341.92 ± 1.99 mg QE/g extract. HPLC-DAD analysis identified 17 phenolic compounds, with hydroxytyrosol being the predominant compound, reaching concentrations of up to 135.16 ± 0.01 mg/g in OMWExt 2. All extracts exhibited strong antioxidant activity, with total antioxidant capacities between 112.79 ± 0.37 and 515.00 ± 1.84 mg EAA/g extract. The FRAP values ranged from 69.78 ± 1.27 mg ET/g extract to 284.73 ± 0.85 mg ET/g, and the optimal IC₅₀ values were 11.99 ± 1.19 μg/mL (DPPH) and 11.91 ± 0.55 μg/mL (ABTS) for OMWExt 2. Antibacterial activity was observed against both Gram-negative (*Escherichia coli* K12, *Pseudomonas aeruginosa* CIP 82.114, and *Klebsiella pneumoniae* CIP A22) and Gram-positive bacteria (*Bacillus subtilis* ATCC 6633 and *Staphylococcus aureus* ATCC 29213), with MIC values ranging from 625 μg/mL to 2.5 mg/mL. Antifungal activity was detected particularly in extracts with high hydroxytyrosol contents, especially OMWExt 2, which presented MIC values between 1.15 and 25 mg/mL against *Candida albicans* ATCC 10231, *Aspergillus flavus* MTCC 9606, *Aspergillus niger* MTCC 282, and *Fusarium oxysporum* MTCC 9913.

## Graphical abstract

**Figure:**
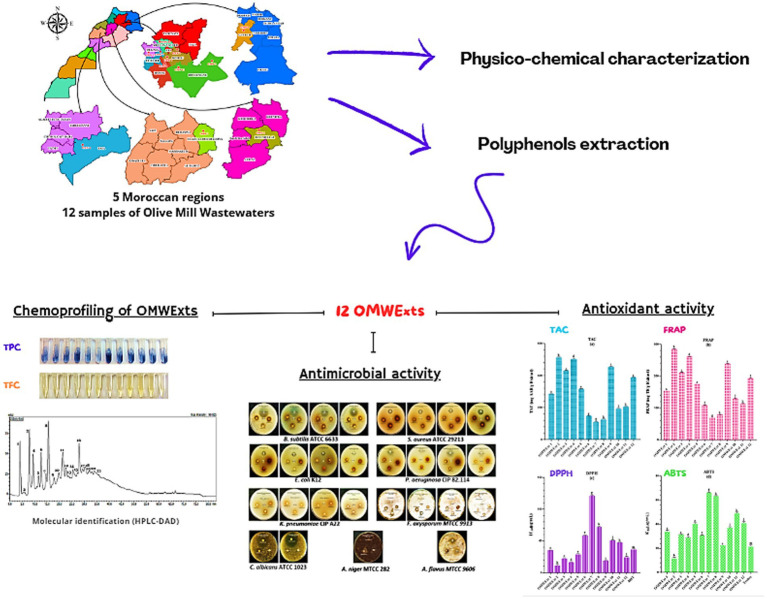

## 1 Introduction

The cultivation of the olive tree represents a fundamental pillar of both the economic and social structure worldwide, particularly in the Mediterranean region (Fountoulakis et al., 2008). Global olive oil production has tripled over the past six decades, reaching 3.27 million tons in the 2019–2020 production cycle (International Olive Oil Council, 2021). In this context, Morocco is a significant contributor, with approximatively 65% of the country’s arboricultural land dedicated to olive trees, which constitute a critical component of its agricultural economy. In 2021–2022, Moroccan olive oil production increased by 21%, reaching 1.96 million tons (Ministry of Agriculture, Maritime Fisheries, Rural Development and Water and Forests, 2022).

The expansion of olive oil production has been marked by a considerable increase in the cultivation of olives on an industrial scale. This growth is directly linked with the generation of substantial quantities of by-products. The main olive oil by-products include olive pomace, olive mill wastewater, olive leaves and olive stones. OMW is the principal liquid waste generated during the olive oil extraction process (El-Emam, 2023). The Mediterranean region produces approximately 30 million cubic meters of OMW every year (Vaz et al., 2024; Chiavola et al., 2014; Al-Qodah et al., 2022). The management of this liquid waste poses a critical environmental challenge because of its high toxicity and problematic effects on ecosystems.

The direct release of this waste into the environment also presents health risks to local communities. In aquatic environments, OMW forms an oily film on the surface, which has been demonstrated to block light penetration and impede oxygen transfer, both of which are vital for the survival of aquatic fauna (Duman et al., 2020; Genç et al., 2020; Görmez et al., 2020). When discharged into the soil, OMW disrupts microbial communities and induces phytotoxic effects on plants, thus leading to the degradation of soil quality and a reduction of the agricultural productivity (Justino et al., 2012). Overall, the ability of OMW to inhibit the growth of certain microorganisms and plants, alters the physicochemical properties of the soil and contaminates groundwater resources (Achak et al., 2009).

The significant ecological impact of OMW is attributed primarily to its high polyphenol content (Shabir et al., 2023; Zagklis et al., 2015; D’Antuono et al., 2014). These polyphenol compounds are particularly resistant to biodegradation, which contributes to the persistence of OMW in the environment. Their accumulation in terrestrial and aquatic ecosystems causes phytotoxic effects, leading to the inhibition of flora and fauna (Farhat et al., 2019). Consequently, the presence of high levels of polyphenols represents a significant environmental challenge, limiting the safe disposal of OMW and requiring appropriate management approaches.

Despite their environmental toxicity, polyphenol compounds are well recognized for their notable biological activities. Numerous studies have reported their antioxidant, cardioprotective, anticancer, anti-inflammatory, and antimicrobial properties (Khwaldia et al., 2022; Bassani, 2015; Li et al., 2014; Fraga et al., 2019). These bioactive properties make OMW a promising and low-cost source of valuable natural compounds for pharmaceutical, cosmetic, and food applications (Rodrigues et al., 2015; Nunes et al., 2016).

The objective of this study was to increse the value of olive mill wastewater (OMW), a significant by-product of the olive oil industry, through a transregional comparative approach. The objective of this study was to investigate the influence of geographical origin on the physicochemical characteristics and polyphenolic composition of OMW samples collected from different olive growing regions of Morocco. Each sample was subjected to a detailed physicochemical analysis, with a particular emphasis on the total polyphenol content. This was followed by the evaluation of antioxidant activity using complementary assays and antimicrobial potential against a broad-spectrum of bacterial and fungal strains. The novelty of this work lies in the comparative assessment of regional variability in bioactive profiles and in establishing a relationship between chemical composition and biological activities. This study demonstrates the potential of OMW as a sustainable and valuable natural source of antioxidant and antimicrobial compounds for future industrial applications by integrating chemical characterization with functional bioactivity evaluation.

## 2 Materials and methods

### 2.1 Study area

**Figure 1:**
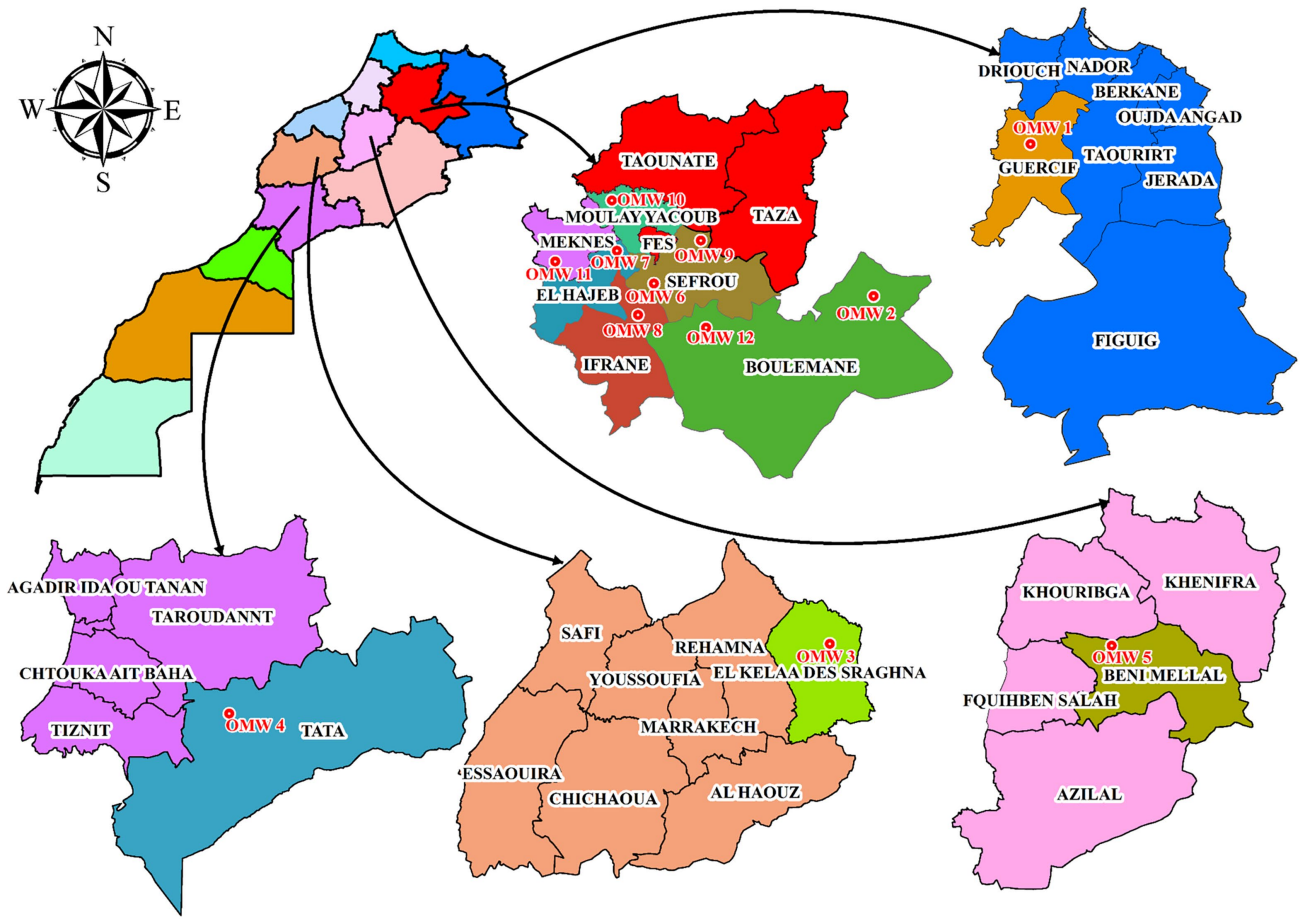
Study area showing the sampling regions of OMW in Morocco.

The study was conducted across five Moroccan regions: Fez-Meknes, Beni Mellal-Khenifra, Marrakech-Safi, the eastern region, and Souss-Massa (Figure 1). During the olive harvest season, between October 2021 and March 2022, 12 OMW samples were collected from industrial mills using a three-phase extraction system. Each sample corresponds to a specific province within the selected region. The geographical and climatic characteristics of each sampling site are presented in Table 1.

**Table 1:** Sites of collection and geographical data.

Sites	Samples	Region	Province	Averge rainfall (mm)	Averge temperature (°C)
Site 1	OMW 1	Oriental	Guercif	300 mm	20 °C
Site 2	OMW 2	Fez- Meknes	Boulmane (Tindite)	120 mm	25 °C
Site 3	OMW 3	Marrakech-Safi	El Kelaa des Sraghna	200 mm	22 °C
Site 4	OMW 4	Souss-Massa	Tata (Tizgui Ida Ou Baloul)	150 mm	21 °C
Site 5	OMW 5	Beni Mellal-Khenifra	Beni Mellal	350 mm	23 °C
Site 6	OMW 6	Fez- Meknes	Sefrou	500 mm	18 °C
Site 7	OMW 7		El Hajeb (M’haya)	500 mm	17 °C
Site 8	OMW 8		Ifrane (Azrou)	900 mm	15 °C
Site 9	OMW 9		Sefrou (Bir Tam Tam)	350 mm	18 °C
Site 10	OMW 10		Moulay Yacoub	600 mm	19 °C
Site 11	OMW 11		Meknes	500 mm	20 °C
Site 12	OMW 12		Boulmane (Outat el haj)	250 mm	19 °C

### 2.2 Physicochemical characterization

The pH and electrical conductivity of the OMW samples were measured via a multiparameter probe. Other parameters including chemical oxygen demand (COD), nitrite (NO_2_^−^), nitrate (NO_3_^−^), ammonium (NH_4_^+^), orthophosphate (PO_4_^3−^) and dry matter (DM) were determined according to the standard methods described by Rodier et al. (2016).

#### 2.2.1 Determination of dry matter

Dry matter (DM) was measured at 105 °C by weighing the sample before and after evaporation in a porcelain crucible. The initial mass (m_0_) of a given volume (V) was recorded, and the sample was then dried until a constant mass (m_1_) was typically achieved after 24 h. The DM content was calculated on the basis of the difference between m₀ and m₁.

$$\mathrm{DM}(\%)=(\frac{\mathrm{Dry}\;\;\text{weight}}{\text{Initial weight}})\times 100$$

#### 2.2.2 Determination of chemical oxygen demand (COD)

The chemical oxygen demand (COD) was determined by oxidizing the organic matter in the sample with an excess of potassium dichromate (K₂Cr₂O₇) in a hot and acidic medium, with mercury sulfate (HgSO₄) and silver sulfate (AgSO₄) serving as catalysts. The samples were heated in a COD reactor, leading to the formation of a yellow-colored complex. This mixture was quantified via colorimetry at 585 nm, and the COD concentration was calculated via a glucose calibration curve.

#### 2.2.3 Quantification of nitrites (NO₂^−^)

The nitrite (NO_2_^−^) content was quantified using Zambelli’s reagent. For each sample, 2 mL of Zambelli’s reagent was added to 50 mL of OMW, and the mixture was allowed to stand for 10 min. Subsequently, 10 mL of ammonia solution was added. The absorbance of the resulting complex was measured at 435 nm using a spectrophotometer. Sodium nitrate was used to construct the calibration curve.

#### 2.2.4 Quantification of nitrates (NO₃^−^)

Nitrate (NO₃^−^) quantification was based on the reduction of nitrate to nitrite, followed by the formation of a yellow-colored complex in the presence of ammonia and sulfosalicylic acid as a catalyst. For this procedure, 10 mL of each sample was mixed with 1 mL of sodium salicylate, and evaporated at 75–80 °C. After cooling, the residue was dissolved in 2 mL of concentrated sulfuric acid. Then, 15 mL of distilled water and 15 mL of sodium potassium tartrate were added. The absorbance of the resulting yellow complex was measured at 415 nm. Potassium nitrate was used to prepare the calibration curve.

#### 2.2.5 Quantification of ammonium (NH₄^+^)

Ammonium (NH₄^+^) was quantified on the basis of its reaction with sodium hypochlorite (NaClO) and phenol (C_6_H_5_OH) in an alkaline medium with sodium nitroprusside (Na_2_[Fe(CN)_5_NO]) serving as a catalyst. This reaction produces indophenol blue, which was quantified by colorimetry. For each sample, 1 mL of sodium nitroprusside solution was added to 20 mL of the sample, followed by the addition of 1 mL of NaClO. Moreover, the complex was incubated in the dark (6 h). The absorbance was measured at 630 nm, and an ammonium chloride calibration curve was used for estimation.

#### 2.2.6 Quantification of orthophosphates (PO₄^3−^)

Orthophosphate (PO₄^3−^) concentrations were quantified by the formation of a blue complex in acidic media. Orthophosphates react with ammonium molybdate and potassium antimonyl tartrate to form a phosphomolybdate complex, which is then reduced by ascorbic acid to produce a blue compound. For this analysis, 1 mL of ascorbic acid was added to 20 mL of OMW and the mixture was homogenized. Then, 4 mL of the complexing reagent (containing ammonium molybdate and potassium antimonyl tartrate) was added. The mixture was incubated for 30 min, and the absorbance was measured at 880 nm. Quantification was established via a dihydrogen phosphate calibration curve.

### 2.3 Polyphenols extraction from olive mill wastewater

Polyphenols extraction from the OMW samples was carried out following the methods of Romeo et al. (2019). First, the lipid fraction was eliminated via the addition of hexane at 1:1 (v/v) ratio for 30 min. This step was repeated three times. The resulting mixture was subsequently transferred to a separating funnel. In addition, the lipid fraction was collected. After that, the polyphenols were extracted for 45 min using ethyl acetate at 1:2 (v/v) ratio, and this process was carried out three times. All the extracted organic phases were combined and centrifuged at 3000 rpm for 5 min. The ethyl acetate was removed under reduced pressure at 40 °C via a rotary evaporator. The concentrated extracts (OMWExts) were finally stored at 4 °C until further use.

### 2.4 Total phenolic content (TPC)

The TPC of the OMWExts samples was quantified by the Folin–Ciocalteu method as described by Singleton et al. (1999). Briefly, 50 μL of each OMWExt was mixed with 500 μL of Folin–Ciocalteu reagent (1:10, v/v), and then incubated for 5 min. Subsequently, 400 μL of 7.5% sodium carbonate solution was added, and the mixture was incubated for 2 h in the dark. The absorbance was measured at 760 nm using a UV/Vis spectrophotometer. All the measurements were performed in triplicate and the TPC was expressed in milligrams of gallic acid equivalent per gram of extract (mg GAE/g Extract). The calibration curve had a correlation coefficient (R^2^) of 0.999.

### 2.5 Total flavonoid content (TFC)

The TFC was quantified via the aluminum chloride (AlCl₃) colorimetric method (Shraim et al., 2021). A total of 500 μL of a 10% AlCl₃ solution was mixed with 500 μL of sample and incubated for 1 h in the dark. The absorbance was measured at 420 nm using a UV/Vis spectrophotometer. The TFC results are expressed as milligrams of quercetin equivalent per gram of extract (mg QE/g extract). The calibration curve (R^2^) has a coefficient of determination of 0.999. All the measurements were performed in triplicate (N = 3).

### 2.6 High-performance liquid chromatography diode array detector analysis (HPLC-DAD)

The qualification and quantification of phenolic compounds in the OMW extracts were performed using high-performance liquid chromatography coupled with a diode array detector (HPLC-DAD), following the method described by Ghomari et al. (2019). The analysis was conducted via a Shimadzu HPLC system equipped with a reverse-phase Wakosil C18HG column (5 μm, 4.6 × 150 mm) maintained at 40 °*C. prior* to analysis, 10 mg of each extract was dissolved in 1 mL of 80% HPLC-grade methanol, filtered through 0.45 mm syringe filters, and prepared for injection. A 20 μL aliquot of each sample was manually injected into the system.

Chromatographic separation was carried out at a flow rate of 1 mL/min via a binary solvent system: solvent A was acidified water (0.2% phosphoric acid), and solvent (B) was a 1:1 (v/v) mixture of HPLC-grade methanol and acetonitrile. A linear gradient program was employed, starting with 96% A and 4% B. initially. Over 40 min, the gradient was gradually shifted to reach 50% A and 50% B. This was followed by a stepwise increase to 40% A and 60% B for 5 min, and then to 100% B (0% A) for 15 min. After the 12-min re-equilibration phase, the system returned to the initial mobile phase composition. Standard phenolic compounds including tannic acid, pyrogallol, gallic acid, catechol, hydroxytyrosol, caffeic acid, kaempferol, tyrosol, protocatechuic acid syringic acid, coumaric acid, ferulic acid, vanillic acid, oleuropein, rutin, quercetin, and rosmarinic acid were prepared in 80% HPLC grade methanol and analyzed under the same chromatographic conditions. The identification and quantification of phenolic compounds in the samples were achieved by comparing the retention times and UV–Vis spectra with those of the authentic standards.

### 2.7 Antioxidant activity

#### 2.7.1 Total antioxidant capacity test (TAC)

The total antioxidant capacity (TAC) of the OMWExts samples was assessed via the ammonium phosphomolybdate assay (El Moussaoui et al., 2019). The reagent solution was prepared by mixing 0.6 M sulfuric acid, 28 mM sodium phosphate and 4 mM ammonium molybdate. The solution was stored in the dark until use. For the assay, 1 mL of the reagent solution was added to 25 μL of each OMWExt sample. The resulting mixture was then incubated at 95 °C for 90 min. The absorbance was measured at 695 nm via a UV/Vis spectrophotometer. All the measurement were performed in triplicate (N = 3), and the TAC results were expressed as milligrams of ascorbic acid equivalent per gram of extract (mg AEA/g extract). The calibration curve showed a linear relationship (R^2^ = 0.999).

#### 2.7.2 Ferric reducing antioxidant power (FRAP assay)

The FRAP assay was conducted according to the protocol described by Bouabid *et al*., with the same modifications (Bouabid et al., 2020). Briefly, three solutions were prepared: acetate buffer (300 mM, pH 3.6), TPTZ solution (10 mM in 40 mM HCl), and FeCl₃ solution (20 mM). Then, 100 μL of each OMWExt sample was combined with 3 mL of FRAP reagent prepared from the three solutions (25 mL of acetate buffer, 2.5 mL of TPTZ solution, and 2.5 mL of FeCl₃ solution). The mixture was incubated in the dark for 30 min, and the absorbance was then measured at 593 nm using a UV/Vis spectrophotometer. All the measurements were performed in triplicate, and the results were expressed as milligrams of Trolox equivalent per gram of extract (mg TE/g extract). The calibration curve showed a strong linear correlation, with a coefficient of determination (R^2^) of 0.999.

#### 2.7.3 Diphenyl-1-picrylhydrazyl (DPPH) assay

The free radical scavenging activity of the OMWExts was assessed via the diphenyl-1-picrylhydrazyl (DPPH) assay, following the method described by Seddoqi et al. (2024), with minors modifications. Each extract was diluted to obtain a range of concentrations; 100, 200, 300, 400 and 500 μg/mL. Then, 100 μL of each diluted sample was mixed with 750 μL of 0.004% methanolic DPPH solution. Butylated hydroxytoluene (BHT) is a standard antioxidant. The reaction mixtures were incubated at room temperature in the dark for 30 min. The absorbance was then measured at 517 nm.

The percentage of inhibition (PI%) of DPPH radicals was calculated via the following equation:

$$\mathrm{PI}\%=(A_{\text{control}}-\frac{A_{\text{sample}}}{A_{\text{control}}})\times 100$$ (1)

Where *A_control_* is the absorbance of the DPPH solution without extract, and *A_sample_* is the absorbance in the presence of the extract.

#### 2.7.4 Radical cation decolorization (ABTS assay)

The antioxidant capacity of the OMWExts was further evaluated using the ABTS radical cation decolorization assay (Tourabi et al., 2023). The ABTS^+^ radical cation was generated by mixing a 7 mM ABTS solution with 2.45 mM potassium persulfate. The resulting mixture was kept in the dark at room temperature for 12–16 h to allow the radical to form. For the test, 150 μL of OMWExts samples at various concentrations (100, 200, 300, 400, and 500 μg/mL) were added to 825 μL of the ABTS^+^ solution. The reaction mixtures were incubated in the dark for 30 min. The absorbance was measured at 734 nm. Trolox was used as the standard antioxidant. The percentage of inhibition (PI%) was calculated using Equation 1.

### 2.8 Antimicrobial activity

The antimicrobial activity of the OMWExts was evaluated against a range of pathogenic microorganisms. The inhibitory effects were tested on Gram-negative bacteria including *Escherichia coli* K12, *Pseudomonas aeruginosa* CIP 82.114 and *Klebsiella pneumoniae* CIP A22, as well as Gram-positive bacteria, namely, *Bacillus subtilis* ATCC 6633 and *Staphylococcus aureus* ATCC 29213. Furthermore, the antifungal efficacy of the OMWExts was assessed against several fungal strains, including *Candida albicans* ATCC 10231, *Aspergillus niger* MTCC 282, *Aspergillus flavus* MTCC 9606, and *Fusarium oxysporum* MTCC 9913.

#### 2.8.1 Disk diffusion method

The antimicrobial activity of the OMWExts was evaluated using the disk diffusion method. Briefly, 100 μL of microbial culture standardized to 0.5 McFarland was mixed with 5 mL of Mueller-Hinton soft agar (0.5% agar) for bacterial strains or Sabouraud soft agar (0.5% agar) for fungal strains. This microbial suspension was then evenly poured onto the surface of Mueller-Hinton agar or Sabouraud agar. Sterile paper discs with a diameter of 6 mm were placed on the solidified agar surface. Each disc was impregnated with OMWExts samples at 10 mg/mL for bacteria and 50 mg/mL for fungi. The plates were incubated at 37 °C for 24 h for bacterial strains and at 30 °C for fungal strains.

After incubation, the diameter of the clear zone of inhibition around each disc was measured in millimeters to assess antimicrobial activity (Ait Assou et al., 2023). Each assay was performed in triplicate. Ampicillin, streptomycin, chloramphenicol and fluconazole served as positive controls, while dimethyl sulfoxide (DMSO) was used as the negative control.

#### 2.8.2 Minimum inhibitory concentration (MIC)

The antimicrobial activity of the OMWExts was further evaluated by determining the minimum inhibitory concentration (MIC), following the guidelines of the National Committee for Clinical Laboratory Standards NCCLS (Ait Assou et al., 2024). The MIC was determined using a 96-well microtiter plate assay. Briefly, Mueller-Hinton broth or Sabouraud broth was mixed with serial twofold dilutions of the OMWExts at concentrations ranging from 0.039 to 10 mg/mL for antibacterial testing, and 0.195 to 50 mg/mL for antifungal testing. Approximately 10^5^ bacterial or fungal cells were added to each well. The contents of each well were mixed gently using a micropipette, and the plates were incubated at 37 °C for bacteria or 30 °C for fungi. Microbial growth was assessed by adding 0.01% resazurin, and the MIC was determined as the lowest concentration of extract that resulted in no visible growth (no pink coloration).

#### 2.8.3 Minimal bactericidal concentration (MBC) and minimal fungicidal concentration (MFC)

To determine the MBC or MFC, aliquots from wells without visible growth were cultured on Mueller-Hinton agar for bacteria or on Sabouraud agar for fungi. After incubation at the appropriate temperature, the MBC or MFC was defined as the lowest concentration of the extract at which no microbial growth was observed, suggesting bactericidal or fungicidal activity, respectively (Boudine et al., 2016; Sarker et al., 2007)

### 2.9 Statistical analysis

All the assays were performed in triplicate, and the results are presented as mean ± standard deviations. The statistical analyses were conducted using GraphPad Prism 8.0.2 (263) software. Differences among groups were evaluated by one-way analysis of variance (ANOVA), followed by Tukey’s multiple comparisons test to assess significance. The correlation coefficient tests were carried out using R 4.2.0 software, and principal component analysis (PCA) was performed viaOriginPro 2025 software (OriginLab Corporation, Northampton, MA, USA).

## 3 Results and discussion

### 3.1 Olive mill wastewaters (OMWs) characterization

The physicochemical characteristics of the OMW samples are summarized in Table 2. All the samples of OMW exhibited acidic pH values, ranging from 3.79 ± 0.17 to 5.54 ± 0.42. This finding is consistent with previous studies that reported similar acidic pH values (Belaqziz et al., 2016; Arabi et al., 2018). The variation in pH values indicates a good agreement with the established data, confirming the acidic nature of the analyzed samples. This acidity is primarily related to the high concentration of total phenolic compounds present in the wastewater. Moreover, the duration of storage in tanks affects acidity levels. During storage, phenolic alcohols undergo polymerization and autoxidation, resulting in the formation of phenolic acids and increased acidity (Paulo and Santos, 2021; Nikiema et al., 2020; Assas et al., 2002).

**Table 2:** Physicochemical characterization of the OMWs.

	pH	Conductivity (ms/cm)	COD (gO_2_/L)	NO_2_^−^ (mg/L)	NO_3_^−^ (mg/L)	NH_4_^+^ (mg/L)	PO_4_^3−^ (mg/L)	DM %
OMW 1	4.34 ± 0.30^a^	11.17 ± 0.76^c^	135.13 ± 0.88^a^	17.86 ± 0.63^a^	80.34 ± 1.30^a^	41.35 ± 2.98^e^	17.16 ± 1.08^a^	19.88 ± 0.89^a,c^
OMW 2	4.25 ± 0.25^a^	10.69 ± 0.50^b,c^	272.80 ± 0.33^b^	53.30 ± 0.44^b^	153.39 ± 0.90^b^	64.44 ± 2.37^f^	52.18 ± 0.34^g^	23.84 ± 0.68^i^
OMW 3	4.75 ± 0.48 ^a^	13.62 ± 0.10^a^	229.02 ± 1.84^c^	37.19 ± 0.96^c^	120.18 ± 1.98^c^	75.73 ± 1.28^b^	33.27 ± 1.27^b,c^	19.07 ± 0.83^a,d^
OMW 4	3.79 ± 0.17^a^	11.83 ± 0.13^c^	331.02 ± 0.69^d^	50.85 ± 0.73^d^	148.33 ± 1.50^d^	55.84 ± 0.74^c^	48.94 ± 0.52^h^	14.01 ± 1.35^e^
OMW 5	4.56 ± 0.45^a^	11.49 ± 0.18^c^	124.80 ± 0.67^e^	72.42 ± 0.32^e^	127.83 ± 0.66^e^	25.88 ± 1.53^a,d^	44.24 ± 0.25^i^	20.70 ± 0.91^a^
OMW 6	4.44 ± 0.47^a^	12.39 ± 0.34^c,d^	209.69 ± 0.38^f^	33.06 ± 1.06^f^	111.66 ± 2.18^f^	51.91 ± 0.43^c,h^	27.54 ± 1.36^j^	11.64 ± 0.70^f^
OMW 7	4.54 ± 0.32^a^	9.63 ± 0.54^b^	184.80 ± 1.00^g^	24.23 ± 0.49^g^	93.47 ± 1.00^g^	28.83 ± 1.13^a^	32.78 ± 0.65^b,d^	9.42 ± 0.69^f,g^
OMW 8	4.46 ± 0.24^a^	9.58 ± 0.40^b^	151.47 ± 1.00^h^	34.95 ± 0.12^h^	115.56 ± 0.25^h^	21.22 ± 1.95^d^	25.11 ± 0.41^k^	15.65 ± 0.41^e^
OMW 9	4.43 ± 0.36^a^	12.64 ± 0.13^a,c,d^	131.13 ± 1.20^i^	26.47 ± 0.76^i^	98.09 ± 1.56^i^	84.82 ± 1.53^g^	33.65 ± 1.42^c,e,f^	17.72 ± 0.82^c,d,h^
OMW 10	5.54 ± 0.42^a^	13.65 ± 0.51^a^	154.24 ± 1.39^h^	30.12 ± 0.24^j^	105.60 ± 0.50^j^	48.48 ± 1.28^h^	34.30 ± 0.19^d,e^	19.90 ± 0.54^a,h^
OMW 11	4.35 ± 0.27^a^	11.59 ± 0.45^c,d^	321.13 ± 0.58^j^	32.29 ± 0.64^f^	110.08 ± 1.32^k^	79.91 ± 2.13^b^	31.54 ± 0.61^b,f^	7.71 ± 0.45^b,g^
OMW 12	4.73 ± 0.34^a^	12.53 ± 0.35^a,c,d^	310.58 ± 1.92^k^	16.32 ± 1.27^a^	77.16 ± 2.61^l^	30.55 ± 0.43^a^	16.35 ± 0.19^a^	6.11 ± 0.86^b^

The values are expressed as the mean ± standard deviations, and the results with the same letter are not significantly different (*p* < 0.05).

All the samples showed high electrical conductivity, ranging from 9.58 ± 0.40 mS/cm for OMW 8 to 13.65 ± 0.51 mS/cm for OMW 10. The observed results are lower than the range reported by Sassi *et al*. (13–41 mS/cm) (Ben Sassi et al., 2006), yet they are comparable to those reported found by El Moudden et al. (2022) (9.5–20.7mS/cm). The high conductivity is associated primarily with the high concentration of dissolved mineral salts, including chlorides and sulfates, as well as the presence of ion-rich organic matter. In addition, the use of commercial salt for olive preservation also contributes to increased electrical conductivity (Elabdouni et al., 2020; Achak et al., 2009).

The analyzed OMW samples also presented a high organic load, which is reflected by the substantial chemical oxygen demand (COD) values. The highest COD levels were observed in OMW 12, 11 and 4, ranging from 310.58 ± 1.92 to 331.0 ± 0.69 gO_2_/L, followed by OMW 6, 3 and 2, with values of 209.69 ± 0.38, 229.02 ± 1.84 and 272.80 ± 0.33, respectively. Moderate COD concentrations were recorded for OMW 8, 10 and 7, ranging from 151.47 ± 1.00 to 184.80 ± 1.00 gO_2_/L. The lowest values were noted in OMW 5, 9, and 1 ranging from 124.80 ± 0.67 to 135.13 ± 0.88 gO_2_/L and the highest COD value was observed in OMW 4, reaching 331.00 ± 0.69 gO₂/L. The findings of the present study demonstrated higher COD values higher compared to than those reported in previous studies (Al-Bsoul et al., 2020; Bottino et al., 2020; Faggiano et al., 2023), but remained lower than those reported in OMW from Spain (Agabo-García et al., 2023). The elevated COD values highlight the substantial organic contamination present in the samples, because of the high abundance of organic matter particularly sugars, organic acids, lipids, and suspended solids which collectively contribute to the overall pollutant load of the wastewater.

The analyzed OMW samples presented important concentrations of several key nutrients and contaminants, including nitrite (NO_2_^−^), nitrate (NO_3_^−^), ammonium (NH_4_^+^), and ortho-phosphate (PO_4_^3−^). Specifically, concentrations ranged from 16.32 ± 1.27 to 72.42 ± 0.32 for NO₂^−^ 77.16 ± 2.61 to 153.39 ± 0.90 mg/L for NO₃^−^, 21.22 ± 1.95 to 84.82 ± 1.53 mg/L for NH₄^+^, and 16.35 ± 0.19 to 52.18 ± 0.34 mg/L for PO₄^3−^. OMW 5 recorded the highest concentration of NO_2_^−^ (72.42 ± 0.32 mg/L), while OMW 2 exhibited the highest levels of both NO_3_^−^ (153.39 ± 0.90 mg/L) and PO_4_^3−^ (52.18 ± 0.34 mg/L). The highest NH₄^+^ concentration was found in OMW 9 (84.82 ± 1.53 mg/L). These concentrations are higher than those reported by Bouknana et al. (2014). Compared with Achak et al. (2009), the concentrations of (NO_2_^−^), (NH_4_^+^), and (PO_4_^3−^) in our samples were greater, whereas the NO₃^−^ concentration was lower. The elevated levels of these nutrients are indicative of a considerable nutrient load in OMW, which engenders environmental concerns such as eutrophication and soil degradation, and effective treatment and management strategies for OMW are paramount.

The dry matter content in our samples, was generally lower than the values reported by Kamal-Eldin (2006). Among all the samples, OMWExt 2 presented the highest dry matter content (23.84 ± 0.68%), whereas OMWExt 11 showed the lowest content (7.71 ± 0.45%). This variation indicates differences in the composition and concentration of solid residuals across samples, which can significantly impact the processing, handling and treatment of OMW.

The study results demonstrated the pollutant loads of these effluents in comparison with those reported in several previous studies. However, some differences are observed, which can be linked to different factors, such as olive variety and maturity, production period, climatic conditions, cultivation practices, geographic region, and the oil extraction process involved. These factors significantly influence the composition and physicochemical characteristics of OMW, resulting in variability in pollutant levels and other measured parameters across different studies.

### 3.2 Chemoprofiling study

**Figure 2:**
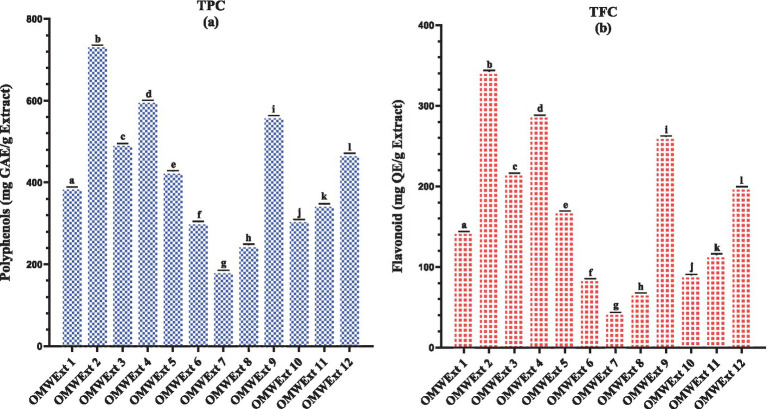
**(a)** Total phenolic content (TPC) and **(b)** total flavonoid content (TFC) of the OMWExts. Tukey’s test showed that results with different letters in the same test are significantly different (*p* < 0.05).

Figure 2a shows the total phenolic content (TPC) of the OMWExts samples. All extracts exhibited a high TPC, with concentrations varying from 183.49 ± 2.18 to 736.56 ± 2.94 mg GAE/g extract. OMWExt 2 recorded the highest phenolic content (736.56 ± 2.94 mg GAE/g extract), whereas OMWExt 7 revealed the lowest phenolic content (183.49 ± 2.18 mg GAE/g extract).

The results for the total flavonoid content (TFC) are summarized in Figure 2b. The TFC values ranged from 43.22 ± 0.42 to 341.92 ± 1.99 mg QE/g extract. OMWExt 2 once more displayed the highest flavonoid concentration (341.92 ± 1.99 mg QE/g extract), whereas OMWExt 7 had the lowest (43.22 ± 0.42 mg QE/g extract).

OMWExt 4 and OMWExt 9 presented the greatest quantities after OMWExt 2, with total phenolic (TPC) contents of 599.84 ± 1.29 mg GAE/g extract and 561.94 ± 1.90 mg GAE/g extract, respectively, and total flavonoid (TFC) contents of 287.89 ± 0.24 mg QE/g extract and 261.30 ± 1.29 mg QE/g extract, respectively.

OMWExt 3, 12, and 5 which showed moderate TPC values of 494.41 ± 0.62, 469.55 ± 1.64, and 426.67 ± 2.24 mg GAE/g extract, respectively, and corresponding TFC values of 215.85 ± 0.42, 198.55 ± 1.12, and 168.86 ± 0.64 mg QE/g extract, respectively. OMWExt 1 and OMWExt 11 recorded TPC values of 387.52 ± 1.24 and 345.68 ± 2.51 mg GAE/g extract, respectively, and TFCs of 143.40 ± 0.49 and 114.41 ± 1.71 mg QE/g extract, respectively. OMWExts values of 10, 6, and 8 are the lowest. The TPC values were 308.39 ± 0.72, 302.80 ± 1.90, and 246.67 ± 2.94 mg GAE/g extract, respectively, while the TFCs were 89.51 ± 1.29, 84.73 ± 0.49, and 67.00 ± 0.88 mg QE/g extract, respectively.

The results indicate significant quantitative variation between all the OMW extract samples. The observed differences in phenolic and flavonoid contents highlight the compositional diversity of OMW due to a variety of environmental and processing factors. According to the study results, phenolic compounds were detected at relatively high concentrations in samples obtained from regions with low levels of rainfall and semiarid climates, where olive trees experience water stress that promotes the biosynthesis of phenolic compounds. In contrast, the lowest concentrations of phenolic compounds were detected in samples from areas with high rainfall and a humid climate. This variability in phenolic composition across regions is attributed not only to climatic conditions but also to edaphic conditions (soil characteristics), olive cultivar., fruit maturity, storage duration of OMW, and the technological processes employed to separate the aqueous (OMW) and oil phases (Mnasri et al., 2017).

Charoenprasert and Mitchell (2012) identified several determinants of phenol content in olives, including cultivar, degree of ripening, and, notably, the methods used for curing and processing table olives. Similarly, Nejad Ebrahimi et al. (2008) attributed variations in chemical composition to the origin of the raw materials, environmental conditions and development stage at the time of collection.

### 3.3 HPLC analysis

The identification and quantification of various components in the different OMWW extracts using HPLC-DAD, are presented in Table 3. A total of 17 phenolic compounds were identified through this analysis, including hydroxycinnamic acids (caffeic acid, coumaric acid, and ferulic acid), hydroxybenzoic acids (gallic acid, protocatechuic acid, syringic acid, vanillic acid), flavonoids (kaempferol, catechol, rutin, and quercetin), phenolic alcohols (tyrosol and hydroxytyrosol) and secoiridoids (oleuropein).

**Table 3:** HPLC-DAD phenolic compounds of OMWExts.

Standards	TR	OMWExt 1	OMWExt 2	OMWExt 3	OMWExt 4	OMWExt 5	OMWExt 6	OMWExt 7	OMWExt 8	OMWExt 9	OMWExt 10	OMWExt 11	OMWExt 12
Tannic acid	2.93	ND	0.35 ± 0.07^a^	0.24 ± 0.05^a^	ND	ND	ND	ND	ND	0.31 ± 0.07^a^	ND	ND	ND
Pyrogallol	3.41	0.14 ± 0.04^a^	ND	0.01 ± 0.07^b^	ND	0.02 ± 0.02^b^	0.34 ± 0.02^c^	1.07 ± 0.01^d^	0.27 ± 0.01^e^	0.38 ± 0.02^c^	0.01 ± 0.03^b^	ND	ND
Gallic acid	5.41	0.31 ± 0.01^a^	1.45 ± 0.09^b^	0.5 ± 0.02^c^	3.66 ± 0.01^d^	0.22 ± 0.03^e^	8.18 ± 0.03^f^	0.94 ± 0.02^g^	2.32 ± 0.06^h^	4.7 ± 0.03^i^	1.6 ± 0.01^j^	0.48 ± 0.01^c^	0.23 ± 0.01^e^
Catechol	6.24	0.07 ± 0.03^a^	18.74 ± 0.09^b^	0.49 ± 0.01^c^	ND	ND	ND	ND	ND	0.38 ± 0.04^d^	ND	0.64 ± 0.01^e^	0.14 ± 0.04^a^
Hydroxytyrosol	7.00	45.93 ± 0.03^a^	135.16 ± 0.01^b^	58.47 ± 0.09^c^	111.85 ± 0.06^d^	46.21 ± 0.05^e^	10.73 ± 0.09^f^	2.57 ± 0.03^g^	10.47 ± 0.02^h^	80.46 ± 0.01^i^	17.71 ± 0.02^j^	28.13 ± 0.02^k^	49.86 ± 0.11^l^
Caffeic acid	8.99	3.47 ± 0.09^a^	1.32 ± 0.11^b^	2.2 ± 0.06^c^	5.4 ± 0.08^d^	7.53 ± 0.07^e^	1.82 ± 0.11^f^	0.84 ± 0.07^g^	3.27 ± 0.04^h^	8.79 ± 0.03^i^	0.8 ± 0.03^g^	1.59 ± 0.04^j^	3.31 ± 0.09^h^
kaempferol	10.68	8.45 ± 0.12^a^	1.09 ± 0.08^b^	9.17 ± 0.09^c^	0.79 ± 0.01^d^	3.8 ± 0.04^e^	12.49 ± 0.03^f^	1.49 ± 0.01^g^	18.49 ± 0.01^h^	1.91 ± 0.05^i^	3.28 ± 0.01^j^	8.81 ± 0.02^k^	10.05 ± 0.05^l^
Tyrosol	11.23	0.26 ± 0.02^a^	2.49 ± 0.05^b^	0.39 ± 0.07^c^	1.08 ± 0.01^d^	11.24 ± 0.01^e^	21.61 ± 0.11^f^	1.48 ± 0.02^g^	15.53 ± 0.02^h^	3.93 ± 0.07^i^	4.85 ± 0.07^j^	0.22 ± 0.08^a^	0.24 ± 0.03^a^
Protocatechuic acid	12.54	0.17 ± 0.08^a^	1.46 ± 0.03^b^	0.12 ± 0.02^a^	4.06 ± 0.02^c^	0.7 ± 0.06^d^	0.88 ± 0.09^e^	0.31 ± 0.04^f^	0.08 ± 0.09^a^	1.05 ± 0.08^g^	2.09 ± 0.05^h^	0.08 ± 0.06^a^	0.06 ± 0.02^a^
Syringic acid	13.25	5.03 ± 0.11^a^	ND	4.63 ± 0.03^b^	0.17 ± 0.03^c^	1.8 ± 0.04^d^	0.94 ± 0.07^d^	0.16 ± 0.03^c^	0.15 ± 0.02^c^	0.39 ± 0.01^f^	0.03 ± 0.05^g^	0.45 ± 0.05^f^	0.28 ± 0.01^h^
Coumaric acid	15.16	0.04 ± 0.04^a^	0.51 ± 0.01^b^	0.07 ± 0.01^a^	2.15 ± 0.01^c^	1.81 ± 0.08^d^	0.41 ± 0.07^e^	0.02 ± 0.02^a^	0.03 ± 0.09^a^	1.54 ± 0.03^f^	2.23 ± 0.04^g^	0.58 ± 0.03^b^	2.74 ± 0.03^h^
Ferulic Acid	17.56	1.21 ± 0.04^a^	2.33 ± 0.01^b^	3.75 ± 0.09^c^	0.56 ± 0.07^d^	0.69 ± 0.04^e^	1.92 ± 0.06^f^	0.05 ± 0.01^g^	0.06 ± 0.07^g^	0.22 ± 0.02^h^	0.56 ± 0.02^d^	1.31 ± 0.02^i^	0.17 ± 0.05^h^
Vanillic acid	18.56	0.15 ± 0.08^a^	0.31 ± 0.02^b^	0.11 ± 0.08^a,c^	0.64 ± 0.01^d^	0.34 ± 0.02^b^	0.74 ± 0.04^e^	0.12 ± 0.04^a,f^	0.8 ± 0.05^e^	0.28 ± 0.06^b^	0.87 ± 0.01^g^	0.04 ± 0.01^c,f^	1.5 ± 0.06^h^
Oleuropein	20.12	ND	0.31 ± 0.09^a^	0.06 ± 0.01^b^	0.97 ± 0.08^c^	0.44 ± 0.01^d^	0.24 ± 0.02^a^	0.07 ± 0.09^b^	0.8 ± 0.03^e^	0.06 ± 0.01^b^	0.12 ± 0.01^b^	ND	ND
Rutin	22.47	8.6 ± 0.02^a^	12.88 ± 0.07^b^	6.92 ± 0.02^c^	35.04 ± 0.05^d^	14.65 ± 0.03^e^	2.74 ± 0.03^f^	0.34 ± 0.07^g^	0.4 ± 0.02^g^	22.00 ± 0.03^h^	5.35 ± 0.02^i^	0.02 ± 0.03^j^	1.61 ± 0.09^k^
Quercetin	27.49	1.51 ± 0.06^a^	1.68 ± 0.1^b^	1.74 ± 0.03^b^	3.97 ± 0.09^c^	4.89 ± 0.04^d^	0.98 ± 0.02^e^	ND	0.91 ± 0.02^e^	3.27 ± 0.02^f^	7.11 ± 0.01^g^	0.22 ± 0.09^h^	2.2 ± 0.1^i^
Rosmarinic acid	32.78	0.41 ± 0.03^a^	0.27 ± 0.02^b^	0.77 ± 0.01^c^	0.63 ± 0.04^d^	0.24 ± 0.03^e,b^	0.07 ± 0.01^f^	ND	0.02 ± 0.01^g,f^	0.2 ± 0.01^h,e^	0.09 ± 0.05^i,f^	0.07 ± 0.01^j,f,g,i^	0.77 ± 0.02^c^

The values are expressed as the mean ± standard deviations (mg/g extract), and the results with the same letter are not significantly different (*p* < 0.05). ND, not detected.

These findings highlight the complex and diverse phenolic profile of OMWExts, confirming their richness in bioactive compounds with potential biofunctional and health promoting properties. The phenolic composition of the various OMW extract samples varied significantly. Hydroxytyrosol emerged as the predominant compound, and was consistently detected in all the samples, with concentrations ranging from 2.57 ± 0.03 to 135.16 ± 0.01 mg/g extract. It was particularly abundant in OMWExt 2 (135.16 ± 0.01 mg/g extract), followed by OMWExt 4 (111.85 ± 0.06 mg/g extract) and OMWExt 9 (80.46 ± 0.01 mg/g extract). Tyrosol, which is another pivotal component of the polyphenolic profile of all the OMW extracts, was present at concentrations ranging from 0.22 ± 0.08 to 21.61 ± 0.11 mg/g extract). The highest levels were found in OMWExt 6 (21.61 ± 0.11 mg/g extract) and OMWExt 8 (15.53 ± 0.02 mg/g extract), which indicates a sample specific variability in phenolic alcohol composition.

In addition, gallic acid and caffeic acid were detected in the samples, with concentrations ranging from 0.23 ± 0.01 to 8.18 ± 0.03 mg/g extract and 0.84 ± 0.07 to 8.79 ± 0.03 mg/g extract, respectively.

The polyphenolic extracts of OMW exhibited a diverse, rich and complex array of phenolic compounds. The flavonoids identified included rutin, kaempferol, and quercetin at various concentrations. Rutin was the most abundant flavonoid, particularly in OMWExt 4, whose content reached 35.04 ± 0.05 mg/g extract. Kaempferol was the second most concentrated compound, with the highest level observed in OMWExt 8 (18.49 ± 0.01 mg/g extract). Quercetin was present in all the samples, although its concentration was the lowest of the three compounds, with a maximum concentration of 7.11 ± 0.01 mg/g extract in OMWExt 10.

Other phenolic compounds, such as catechol, tannic acid and pyrogallol, were identified only at negligible concentrations. Tannic acid was identified in only three samples: OMWExts 2, 3 and 9. In contrast, catechol was detected across all 12 samples, with a notable presence, particularly in OMWExts 1, 2, 3, 9, 11 and 12. Pyrogallol was absent in OMWExts 2, 4, 11 and 12, but was present, even at low concentrations, in the remaining samples. The complexity of OMW phenolic profiles is highlighted by the varying presence and concentrations of these compounds, which underscores the importance of the tailored extraction and purification processes employed to optimize the recovery of target phenolic compounds for specific industrial or therapeutic applications.

### 3.4 Antioxidant activity

**Figure 3:**
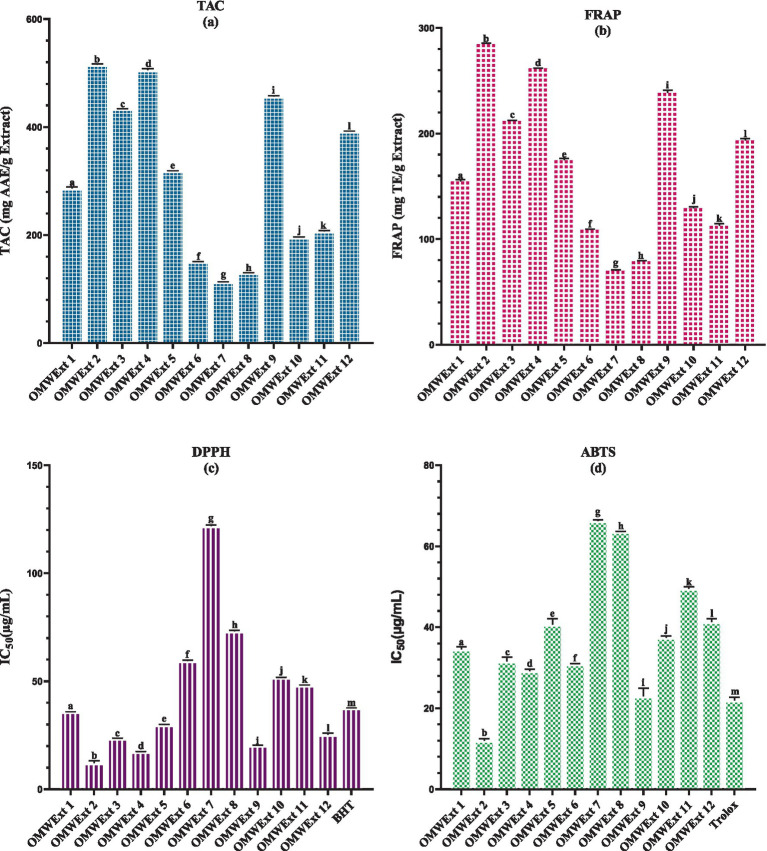
**(a)** Total antioxidant activity (TAC), **(b)** ferric reducing antioxidant power (FRAP assay), **(c)** radical scavenging activity (DPPH assay), **(d)** radical scavenging activity (ABTS assay). Tukey’s multiple range test revealed that the results with different letters in the same test are significantly different (*p* < 0.05).

The antioxidant activity of the OMWExts was assessed via four common assays: total antioxidant capacity (TAC), plasma reducing power (PRP), and DPPH and ABTS radical scavenging activity. All the OMWExts samples presented high antioxidant activity, although notable differences were detected among them (Figure 3).

The TAC values ranged from 112.79 ± 0.37 to 515.00 ± 1.84 mg EAA/g extract, indicating the potent antioxidant potential of these extracts (Figure 3a). The highest antioxidant activity was recorded in OMWExt 2 (515.00 ± 1.84 mg EAA/g extract) and OMWExt 4 (505.07 ± 2.94 mg EAA/g extract), followed by OMWExt 9, OMWExt 3 and OMWExt 12, with values of 456.18 ± 1.84, 433.38 ± 0.37 and 391.47 ± 1.10 mg EAA/g extract, respectively. A relatively moderate antioxidant capacity was observed in OMWExt 1 and OMWExt 5, with 318.31 ± 0.74 and 285.96 ± 2.94 mg EAA/g extract, respectively. These were followed by OMWExt 11 (206.54 ± 1.47 mg EAA/g extract), OMWExt 10 (195.15 ± 1.10 mg EAA/g extract) and OMWExt 6 (150.29 ± 0.37 mg EAA/g extract). The lowest activities were observed in OMWExt 8 (129.71 ± 0.37 mg EAA/g extract) and OMWExt 7 (112.79 ± 0.37 mg EAA/g extract).

The FRAP assay demonstrated that all OMWExts samples exhibit a reducing power, although their antioxidant capacities varied significantly (Figure 3b). Among the samples, OMWExts 2, 4, 9 and 3 demonstrated the highest FRAP values. OMWExt 2 had the strongest reducing ability, with a value of 284.73 ± 0.85 mg ET/g extract, followed by OMWExt 4 (261.45 ± 0.42 mg ET/g extract), OMWExt 9 (238.41 ± 2.58 mg ET/g extract) and OMWExt 3 (211.69 ± 0.74 mg ET/g extract). OMWExt 12 and OMWExt 5 presented moderate reducing capacities, reaching 193.31 ± 1.95 and 174.68 ± 1.85 mg ET/g extract, respectively. In contrast, OMWExt 1, 10, 11, and 6 showed lower FRAP values, with 154.58 ± 1.70, 129.09 ± 1.70, 112.67 ± 1.85 and 108.75 ± 0.74 mg ET/g extract, respectively. Finally, the lowest activities were observed in OMWExt 8 and OMWExt 7, with values of 79.09 ± 0.42 and 69.78 ± 1.27 mg ET/g extract, respectively.

All the extracts were able to scavenge the DPPH radical, with IC_50_ values ranging from 11.99 ± 1.19 to 121.74 ± 0.53 μg/mL (Figure 3c). Notably, OMWExts 2, 4, 9, 3, 12 and 5 presented IC_50_ values lower than those of the standard antioxidant BHT (37.5 ± 0.13 μg/mL), indicating stronger radical scavenging activity. OMWExt 2 showed the most potent activity, with an IC₅₀ value of 11.99 ± 1.19 μg/mL, followed by OMWExt 4, (17.27 ± 0.09 μg/mL), 9 (20.17 ± 0.11 μg/mL), 3 (23.38 ± 0.20 μg/mL), 12 (25.15 ± 0.88 μg/mL), and OMWExt 5 (29.63 ± 0.41 μg/mL). In contrast, OMWExts 1, 11, 10, 6, 8, and 7 displayed higher IC_50_ values than did BHT, with respective values of 35.76 ± 0.10, 47.99 ± 0.27, 51.63 ± 0.03, 59.29 ± 0.38, 73.04 ± 0.44 and 121.74 ± 0.53 μg/mL, respectively. The results underscore the strong antioxidant potential of all the OMW extracts, highlighting their efficacy in neutralizing free radicals and suggesting their promising application as natural antioxidant agents.

The IC₅₀ values for the ABTS radical scavenging assay ranged from 11.91 ± 0.55 to 66.18 ± 0.31 μg/mL. Compared with the standard antioxidant Trolox (21.87 μg/mL), all the OMWExts samples exhibited higher IC₅₀ values, except for OMWExt 2 which presented the strongest antioxidant activity, with the lowest IC₅₀ value of 11.91 ± 0.55 μg/mL, suggesting its potential as a natural antioxidant. OMWExts 9, 4, 6 and 3 demonstrated high antioxidant activity, with IC₅₀ values of 22.89 ± 2.01, 29.12 ± 0.56, 30.84 ± 0.14, and 31.52 ± 1.15 μg/mL, respectively. Moderate antioxidant capacities were observed in OMWExts 1, 10, 5, 12, and 11, with IC₅₀ values ranging from 34.48 ± 0.69 to 49.45 ± 0.55 μg/mL. Finally, the lowest activities were recorded in OMWExts 7 and 8, with IC₅₀ values of 63.53 ± 0.99 and 66.18 ± 0.31 μg/mL, respectively (Figure 3d).

Overall, HPLC analysis revealed that the high phenolic content of the OMWExts that strongly aligns with their significant performance in antioxidant assays, which highlights their promising potential for applications requiring potent antioxidant properties. Also, Farag *et al*, reported a significant association between antioxidant efficacy and the chemical composition of phenolic compounds (Farag et al., 2003). According to another study, the specific phenolic profile plays a more decisive role in determining antioxidant activity than does the overall phenolic content (Bouaziz et al., 2005). Our results on the antioxidant activity of the OMW phenolic extracts align with those of previous studies, confirming their strong antioxidant potential (El-Abbassi et al., 2012; Kachouri and Hamdi, 2004; Di Mauro et al., 2017).

OMWExt 2 demonstrated the highest antioxidant activity among all the OMW extracts in the four assays. HPLC analysis revealed that OMWExt 2 contained the greatest concentration of phenolic compounds among the extracts analyzed. Specifically, it comprises hydroxytyrosol (13.5%) and catechol (1.8%), which are the most abundant compounds detected. The antioxidant capacity mainly depends on the catechol structure and the number of hydroxyl groups present (Xie et al., 2015). Catechol, because of its two adjacent hydroxyl groups on the aromatic ring, contributes hydrogen atoms to neutralize free radicals and stabilize the resulting radical through electron delocalization, thereby promoting antioxidant activity (Meng et al., 2015). Hydroxytyrosol is a catechol derivative predominantly present in this extract, that combines this catechol structure with an additional hydroxyl group on its side chain, further enhancing its antioxidant properties (Andrés et al., 2023).

The observed antioxidant potential can also be linked to the synergistic effects between the phenolic compounds present in the extracts, including phenolic acids and flavonoids. Among the phenolic acids, caffeic acid is characterized by its notably high antioxidant activity, unlike *p*-coumaric and syringic acids, which are less effective because they have only one hydroxyl group (Fki et al., 2005). Flavonoids, including rutin and quercetin, contain three aromatic rings and several hydroxyl groups, contributing significantly to the overall antioxidant power (Suárez et al., 2009). The synergistic interactions among the different compounds play a pivotal role in improving their overall effectiveness.

### 3.5 Antimicrobial activity

#### 3.5.1 Disk diffusion method

The polyphenolic extracts exhibited significant antibacterial activity against all five tested bacterial strains, although the inhibition intensity varied depending on the extract. In contrast, the fungal species (*C. albicans*, *A. niger*, and *A. flavus*) were generally resistant to the treatments, except for OMWExt 2, which showed antifungal activity. Additionally, OMWExt 4 demonstrated a specific inhibitory effect on *C. albicans*. The negative control (DMSO) had no antimicrobial effect, whereas the positive controls were effective against all the tested microorganisms (Table 4).

**Table 4:** Antimicrobial activity of the OMWExts [diameter of the inhibition zone (mm)].

	Diameter of the inhibition zone (mm)								
	Antibacterial activity					Antifungal activity			
	*B. subtilis*	*S. aureus*	*E. coli*	*P. aeruginosa*	*K. pneumoniae*	*C. albicans*	*A. niger*	*A. flavus*	*F. oxysporum*
OMWExt 1	10.00 ± 0.71^b^	15.00 ± 0.71^a^	12.00 ± 0.35^a,b^	10.25 ± 0.35^a,b^	10.75 ± 0.35^a^	NZ	NZ	NZ	8.75 ± 0.71^a,b^
OMWExt 2	11.25 ± 0.35^a^	16.88 ± 0.53^b^	15.13 ± 0.18^f^	12.38 ± 0.18^c^	13.13 ± 0.18^g,h,i^	11.63 ± 0.18^a^	9.38 ± 0.53	9.50 ± 1.41	15.00 ± 0.35^h^
OMWExt 3	12.88 ± 0.18^e^	15.38 ± 1.24^a^	11.25 ± 0.35^a^	10.13 ± 0.53^a,b,e^	13.88 ± 0.53^b,g^	NZ	NZ	NZ	10.00 ± 1.41^a,d^
OMWExt 4	15.13 ± 0.53^c^	11.13 ± 0.18^d^	11.13 ± 0.53^a^	12.50 ± 0.71^c^	12.13 ± 0.53^c,h^	8.50 ± 0.35^b^	NZ	NZ	12.13 ± 0.53^f^
OMWExt 5	15.00 ± 0.71^c^	10.63 ± 0.18^d^	13.50 ± 0.71^c^	9.13 ± 0.53^a,d^	12.25 ± 1.06^c,i^	NZ	NZ	NZ	9.38 ± 0.88^a,c,d,e^
OMWExt 6	11.38 ± 0.18^a^	13.00 ± 0.71^c^	9.25 ± 0.35^d,e^	8.25 ± 0.35^d,f^	10.13 ± 0.53^a,f^	NZ	NZ	NZ	7.75 ± 0.71^b,g^
OMWExt 7	11.13 ± 0.18^a^	12.75 ± 0.35^c^	8.13 ± 0.53^d^	9.38 ± 0.18^a,f,g,h^	7.50 ± 0.71^d^	NZ	NZ	NZ	8.00 ± 0.35^b,c,g^
OMWExt 8	11.00 ± 0.71^a^	13.75 ± 1.06^c^	10.50 ± 0.71^a,e^	9.00 ± 1.06^d,e,g^	7.25 ± 0.35^d^	NZ	NZ	NZ	8.13 ± 0.18^b,c,g^
OMWExt 9	10.38 ± 0.88^b^	16.13 ± 0.88^a,b^	11.00 ± 0.71^a^	12.25 ± 0.35^c^	12.38 ± 0.53^c^	NZ	NZ	NZ	10.50 ± 0.35^d,e^
OMWExt 10	9.13 ± 0.17^f^	13.25 ± 0.71^c^	10.88 ± 1.24^a^	10.38 ± 0.88^b,h^	10.25 ± 0.35^e,f^	NZ	NZ	NZ	7.88 ± 0.18^b,g^
OMWExt 11	8.35 ± 0.35^d^	15.25 ± 0.35^a^	10.75 ± 1.77^a^	12.38 ± 0.53^c^	10.25 ± 0.35^a,e^	NZ	NZ	NZ	8.00 ± 0.71^b,c,g^
OMWExt 12	8.50 ± 0.71^d^	15.75 ± 0.71^a,b^	12.88 ± 0.18^b,c^	12.63 ± 0.18^c^	8.13 ± 0.53^d^	NZ	NZ	NZ	13.13 ± 0.88^f^
Gentamicin	27.25 ± 0.35^g^	–	–	–	–	–	–	–	–
Ampicillin	–	19.25 ± 0.35^e^	13.00 ± 0.71^b,c^	–	–	–	–	–	–
Streptomycin	–	–	–	19.25 ± 0.35	–	–	–	–	–
Chloramphenicol	–	–	–	–	14.75 ± 0.71^b^	–	–	–	–
Fluconazole	–	–	–	–	–	34 ± 1.41^c^	NZ	NZ	NZ

The values are represented as the mean ± standard deviations, and the results with the same letter are not significantly different (*p* < 0.05). NZ: no zone of inhibition, (−): not tested.

Regarding antibacterial potency, *B. subtilis* was the most susceptible strain, displaying the widest inhibition zones, particularly with OMWExt 4 (15.13 ± 0.53 mm) and OMWExt 5 (15.00 ± 0.71 mm), which were identified as the most active extracts against this bacterium. OMWExts 2, 3, 6, 7, and 8 demonstrated moderate activity, with inhibition diameters ranging from 11.00 ± 0.71 to 12.88 ± 0.18 mm. In comparison, OMWExts 1, 9, 10, 11, and 12 exhibited relatively lower antibacterial effects, producing inhibition zones between 8.35 ± 0.35 and 10.00 ± 0.71 mm.

*S. aureus* showed the highest sensitivity, with zones of inhibition ranging from 10.63 ± 0.18 to 16.88 ± 0.53 mm. The OMWExts 1, 2, 3, 9, 11 and 12 had the strongest inhibitory effects, with inhibition zones ranging between 15.00 ± 0.71 and 16.88 ± 0.53 mm. In addition, OMWExt 2 exhibited the largest inhibition zone (16.88 ± 0.53 mm), whereas OMWExts 4, 5, 7, 8, and 10 induced moderate antibacterial activity, with inhibition zones between 10.63 ± 0.18 and 13.75 ± 1.06 mm.

The inhibition zones of the extracts against *E. coli* varied from 8.13 ± 0.53 to 15.13 ± 0.18 mm, with OMWExt 2 having the highest inhibitory activity (15.13 ± 0.18 mm) and OMWExts 1, 5, and 12 showing moderate antibacterial activity, with inhibition zones ranging from 12.00 ± 0.35 to 13.50 ± 0.71 mm. OMWExts 3, 4, 6, 7, 8, 9, 10, and 11 had lower activity, with inhibition zones ranging from 8.13 ± 0.53 to 11.25 ± 0.35 mm. OMWExts 2, 4, 9, 11, and 12 demonstrated the highest activity against *P. aeruginosa*, with a 12 mm inhibition zone for all of them. The remaining extracts (OMWExts 1, 3, 5, 6, 7, 8 and 10) presented smaller inhibition zones, ranging from 8.25 ± 0.35 to 10.38 ± 0.88 mm, and the OMWExt 6 had the lowest value (8.25 ± 0.35 mm). For *K. pneumoniae*, OMWExts 2, 3, 4, 5 and 9 demonstrated significant efficacy, with inhibition zones ranging from 12.13 ± 0.53 to 13.88 ± 0.53 mm. OMWExts 1, 6, 10 and 11 exhibited moderate activity (10 mm), however OMWExts 7, 8 and 12 showed weaker inhibition, with zones measuring 7.25 ± 0.35 to 8.13 ± 0.53 mm. The reference antibiotics exhibited marked antibacterial activity, generating inhibition zones of 27.25 ± 0.35 mm against *B. subtilis*, 19.25 ± 0.35 mm against *S. aureus*, 13 ± 0.71 mm against *E. coli*, 19.25 ± 0.35 mm against *P. aeruginosa* and 14.75 ± 0.71 mm against *K. pneumoniae*.

Among the tested fungal species, *F. oxysporum* was identified as the most sensitive strain. OMWExts 2, 4 and 12 produced inhibition zones ranging from 12.13 ± 0.53 to 15.00 ± 0.35 mm. The remaining extracts (OMWExts 1, 3, 5, 6, 7, 8, 9, 10 and 11) demonstrated smaller inhibition zones between 7.75 ± 0.71 and 10.50 ± 0.35 mm. In contrast, the fungal strains *C. albicans*, *A. niger* and *A. flavus* were resistant to all the extracts except OMWExt 2, which presented inhibition zones of 11.63 ± 0.18 mm, 9.38 ± 0.53 mm and 9.50 ± 1.41 mm, respectively. Furthermore, OMWExt 4 inhibited *C. albicans*, resulting in an inhibition zone of 8.50 ± 0.35 mm. Fluconazole, which was used as the positive control, generated an inhibition zone of 34 ± 1.41 mm against *C. albicans*, whereas no inhibition was detected against *A. flavus*, *A. niger* or *F. oxysporum* in the disk diffusion assay.

#### 3.5.2 MICs of the OMWExts

The MIC values obtained from the microdilution assay are summarized in Table 5. The MICs of all the extracts ranged from 0.625 to 2.5 mg/mL, depending on both the extract (OMWExts) and the bacterial strain. OMWExt 2 exhibited the highest antibacterial activity, with an MIC of 0.625 mg/mL against the Gram-positive strains *B. subtilis* and *S. aureus*. OMWExts 4, 5, 6, 9, and 12 reveled MIC values of 1.25 mg/mL. All remaining extracts displayed MIC values of 2.5 mg/mL against these strains. Among the Gram-negative bacteria, *E. coli* was sensitive to OMWExts 2, 5, 9 and 12, which all demonstrated an MIC of 1.25 mg/mL, whereas the other extracts presented MICs of 2.5 mg/mL. Furthermore, *P. aeruginosa* was inhibited by OMWExts 2 and 4, with MIC values of 0.625 mg/mL. OMWExt 12 had an MIC of 1.25 mg/mL, whereas all other extracts showed a lower activity (MIC = 2.5 mg/mL). OMWExts 2 and 4 again had the strongest activity (MIC = 1.25 mg/mL) against *K. pneumoniae,* while all other extracts exhibited MIC values of 2.5 mg/mL. The antibiotic controls demonstrated substantially greater activity, with MIC values of 2 μg/mL against *B. subtilis*, and *S. aureus*, and 4 μg/mL against *E. coli*, *P. aeruginosa*, and 6 μg/mL *K. pneumoniae*.

**Table 5:** MIC of the OMWExts (mg/mL).

	MIC (mg/mL)								
	*B. subtilis*	*S. aureus*	*E. coli*	*P. aeruginosa*	*K. pneumoniae*	*C. albicans*	*A. flavus*	*A. niger*	*F. oxysporum*
OMWExt 1	2.5	2.5	2.5	2.5	2.5	3.12	12.5	25	6.25
OMWExt 2	0.625	0.625	1.25	0.625	1.25	1.56	3.12	6.25	1.56
OMWExt 3	2.5	2.5	2.5	2.5	2.5	3.12	12.5	25	3.12
OMWExt 4	1.25	1.25	2.5	0.625	1.25	3.12	12.5	6.25	3.12
OMWExt 5	1.25	1.25	1.25	2.5	2.5	3.12	12.5	25	3.12
OMWExt 6	1.25	1.25	2.5	2.5	2.5	6.25	12.5	25	3.12
OMWExt 7	2.5	2.5	2.5	2.5	2.5	6.25	25	25	6.25
OMWExt 8	2.5	2.5	2.5	2.5	2.5	6.25	25	25	6.25
OMWExt 9	1.25	1.25	1.25	2.5	2.5	3.12	3.12	6.25	3.12
OMWExt 10	2.5	2.5	2.5	2.5	2.5	6.25	12.5	25	3.12
OMWExt 11	2.5	2.5	2.5	2.5	2.5	6.25	12.5	25	3.12
OMWExt 12	1.25	1.25	1.25	1.25	2.5	3.12	3.12	25	3.12
Gentamicin	0.002	–	-	–	–	–	–	–	–
Ampicillin	–	0.002	0.004	–	–	–	–	–	–
Streptomycin	–	–	–	0.004	–	–	–	–	–
Chloramphenicol	–	–	–		0.006	–	–	–	–
Fluconazole	–	–	–	–	–	0.008	0.025	0.02	0.05

(−) not tested.

The MIC values of the extracts against fungal strains ranged from 1.56 to 25 mg/mL. OMWExt 2 retained the strongest antifungal activity, with an MIC of 1.56 mg/mL against *C. albicans*. OMWExts 1, 3, 4, 5, 9, and 12 had moderate activity against this strain (MIC = 3.12 mg/mL), whereas 6.25 mg/mL was observed for OMWExts 6, 7, 8, 10, and 11. Against *A. flavus*, OMWExts 2, 9 and 12 exhibited the most optimal activity (3.12 mg/mL). All extracts displayed an MIC value of 12.5 mg/mL, except for OMWExts 7 and 8, which indicated 25 mg/mL. OMWExts 2, 4, and 9 were active against *A. niger* (MIC of 6.25 mg/mL), whereas all remaining extracts had MIC values of 25 mg/mL. For *F. oxysporum*, OMWExt 2 again demonstrated the highest inhibitory effect (MIC = 1.56 mg/mL). OMWExts 3, 4, 5, 6, 9, 10, 11, and 12 demonstrated moderate activity (MIC of 3.12 mg/mL), while OMWExts 1, 7, and 8 revealed the lowest activity (6.25 mg/mL). The MIC values of fluconazole, used as the reference drug, were 8 μg/mL against *C. albicans*, 25 μg/mL against *A. flavus*, 20 μg/mL against *A. niger* and 50 μg/mL against *F. oxysporum*.

#### 3.5.3 MBC and MFC of the OMW extracts

All OMWExts samples demonstrated both bactericidal activities, and not all extracts demonstrated fungicidal activity, as reflected by their MBC and MFC values (Table 6). For Gram-positive bacteria, the MBC of all extracts was 5 mg/mL, except for OMWExt 2, which had a lower MBC of 2.5 mg/mL. Among the Gram-negative strains, OMWExts 2, 4, 9 and 12 displayed an MBC of 5 mg/mL against *E. coli*, while the remaining extracts exhibited MBC values of 10 mg/mL. With respect to *P. aeruginosa*, all the extracts had MBC values of 10 mg/mL, except for OMWExts 2 and 9 (MIC = 5 mg/mL). Similarly, for *K. pneumoniae*, most extracts showed MBCs of 10 mg/mL, whereas OMWExts 2, 4, 9 and 12 recorded lower MBCs of 5 mg/mL. In comparison, the reference antibiotics showed high efficacy, with MBC values of 2 μg/mL against *B. subtilis* ATCC 6633 and *S. aureus*, 4 μg/mL against *E. coli* and *P. aeruginosa*, and 6 μg/mL against *K. pneumoniae*.

**Table 6:** MBC and MFC of the OMWExts (mg/mL).

	MBC and MFC (mg/mL)								
	*B. subtilis*	*S. aureus*	*E. coli*	*P. aeruginosa*	*K. pneumoniae*	*C. albicans*	*A. flavus*	*A. niger*	*F. oxysporum*
OMWExt 1	5	5	10	10	10	25	25	>50	25
OMWExt 2	2.5	2.5	5	5	5	6.25	12.5	25	12.5
OMWExt 3	5	5	10	10	10	25	25	>50	25
OMWExt 4	5	5	5	10	5	25	25	25	25
OMWExt 5	5	5	10	10	10	25	25	>50	25
OMWExt 6	5	5	10	10	10	>50	25	>50	25
OMWExt 7	5	5	10	10	10	>50	>50	>50	50
OMWExt 8	5	5	10	10	10	>50	>50	>50	50
OMWExt 9	5	5	5	5	5	25	25	25	25
OMWExt 10	5	5	10	10	10	>50	25	>50	25
OMWExt 11	5	5	10	10	10	>50	25	>50	25
OMWExt 12	5	5	5	10	5	25	25	>50	25
Gentamicin	0.002	–	–	–	–	–	–	–	–
Ampicillin	–	0.002	0.004	–	–	–	–	–	–
Streptomycin	–	–	–	0.004	–	–	–	–	–
Chloramphenicol	–	–	–		0.006	–	–	–	–
Fluconazole	–	–	–	–	–	0.008	0.025	0.02	0.05

(−) not tested.

All OMWExts samples showed MFCs ranging from 6.25 to 50 mg/mL against the four fungal strains. OMWExt 2, which demonstrated stronger antifungal activity, with an MFC of 6.25 mg/mL against *C. albicans*. OMWExts 1, 3, 4, 5, 9, and 12 presented a moderate MFC of 25 mg/mL. For all other extracts, the MFC was greater than 50 mg/mL. For *A. flavus*, OMWExt 2 exhibited the greatest activity at a concentration of 12.5 mg/mL, and the other extracts demonstrated activity at 25 mg/mL, except for OMWExts 7 and 8, which had MFCs greater than 50 mg/mL. The MFC for *A. niger* was greater than 50 mg/mL, while for OMWExts 2, 4 and 9, the MFC was determined to be 25 mg/mL. OMWExts 7 and 8 exhibited higher MFC values of 50 mg/mL. The MFCs against *F. oxysporum* were 12.5 mg/mL for OMWExt 2, 50 mg/mL for OMWExts 7 and 8, and 25 mg/mL for all other extracts.

The 12 OMWExts samples clearly presented broad-spectrum antimicrobial activity against nine pathogenic microorganisms. OMWExt 2 demonstrated the most remarkable efficacy, with large zones of inhibition and the lowest MIC values across all the pathogens and was the only extract that inhibited the growth of all the fungal strains.

This enhanced antimicrobial activity is associated with the high concentration of phenolic compounds, particularly hydroxytyrosol. These findings indicate that OMWExt 2 is a highly potent natural antimicrobial agent capable of targeting both Gram-positive and Gram-negative bacteria, as well as fungal strains. Furthermore, OMWExts 3, 4, 9, and 12 also demonstrated strong antimicrobial activity, supporting the potential of these phenolic extracts as broad-spectrum antimicrobial agents. In contrast, OMWExts 10 and OMWExt 11 exhibited moderate antibacterial activity, although their efficacy varied depending on the microorganism, which suggests their selective antimicrobial potential. OMWExts 7 and 8 demonstrated the lowest activity, characterized by narrow zones of inhibition and high MIC values, because of their reduced content of active phenolic compounds.

The differences in antibacterial potential observed between Gram-negative and Gram-positive bacteria can be attributed to variations in their cell wall structure. Gram-negative bacteria possess an outer membrane that is rich in lipopolysaccharides, which act as a permeability barrier and increase their resistance to antimicrobial agents. Despite this structural barrier, all the extracts showed significant antimicrobial activity against Gram-negative strains, because of the hydrophobic nature of the biophenols present in olive by-products, which enables interaction with and disruption of the bacterial outer membrane (Obied et al., 2007).

Our results aligned with those reported by Yakhlef et al. (2018) who reported that the OMW extracts with the highest phenolic content exhibited the strongest antibacterial effects. The antimicrobial potential of phenolic extracts is strongly linked to their composition and differences in molecular structure, and the functional groups affect their interaction with bacterial cells (Roila et al., 2016). Similarly, Yangui et al. (2010) demonstrated that hydroxytyrosol, tyrosol and luteolin have a significant antimicrobial activity against Gram-positive bacteria. Bisignano et al. (1999) reported that hydroxytyrosol and oleuropein, which are major phenolic compounds in olives, exhibited cytotoxic activity against clinical strains, particularly *S. aureus.*

Moreover, Obied et al. (2007) revealed that the antibacterial activity of olive mill by-products toward both Gram-positive and Gram-negative bacteria, as well as yeasts, was attributed primarily to hydroxytyrosol. In support of these findings, Australian OMW extract demonstrated inhibitory activity against four pathogenic bacteria (*S. aureus, B. subtilis, E. coli,* and *P. aeruginosa*) at a concentration of 5 mg per plate in a disk diffusion assay. The antimicrobial capacity was further enhanced by the work of Tafesh *et al.,* who further confirmed the antimicrobial potential of hydroxytyrosol, which inhibited *Streptococcus pyogenes, S. aureus, E. coli and Klebsiella* (K.) in a broth microdilution test (Tafesh et al., 2011).

For the disk-diffusion assay performed on *C. albicans*, *A. niger* and *A. flavus*, no inhibition zones were observed for most of the extracts or for fluconazole, except for OMWExts 2 and 4. These results indicate that, although the disk diffusion method, is useful for rapid preliminary screening, it may not accurately reflect the susceptibility of certain fungal strains, particularly those possessing intrinsic or adaptive resistance mechanisms. Therefore, integrating disc diffusion results with MIC/MFC data is essential to obtain a more reliable evaluation of antifungal susceptibility.

Some of our samples (OMWExts 6, 7, 8, 10 and 11) corroborate the lack of antifungal activity previously reported by Obied *et al*, who revealed no inhibition of *C. albicans* or *A. niger*. Conversely, extracts with relatively higher hydroxytyrosol concetrations showed marked antifungal activity, suggesting that the presence and concentration of specific phenolic compounds, such as hydroxytyrosol, play a key role in the antifungal efficacy (Obied et al., 2007). Similarly, Zoric et al. (2013) reported MFC values ranging from 97.6 μg/mL to 6.25 mg/mL, highlighting the strong antifungal efficacy of hydroxytyrosol against *C. albicans*.

Gourama et al. (1989) investigated the antifungal potential of individual phenolic compounds, tyrosol, caffeic acid and oleuropein, and observed inhibitory effects against *Aspergillus* and *Penicillium* species derived from olives. Furthermore, Chaves-López et al. (2015) evaluated the antifungal activity of phenolic fractions from olive mills and reported the inhibition of several fungal species, including *Penicillium expansum* DSMZ 1282 and *Penicillium verrucosum* DSMZ 12. These studies further revealed that OMWW inhibited multiple fungal species, such as *Aspergillus clavatus* DSMZ 816, *Eurotium amstelodami* MS10, and *Cladosporium cladosporioides* MS1, reinforcing its potential as a natural source for the development of new antifungal agents.

Olive mill wastewater extracts have been shown to exhibit significant antimicrobial activity, primarily due to their high concentration of polyphenolic compounds. Numerous studies have reported that the phenolic compounds present in polyphenolic extracts have significant antimicrobial effects, primarily due to their ability to disrupt cell membranes, increase permeability and compromise integrity, ultimately resulting in cellular dysfunction and microbial death (Qi et al., 2022; Keyvani-Ghamsari et al., 2023; Pavlíková, 2023).

In the present study, OMWExts 2, 4 and 9 exhibited particularly high activity. Their phenolic profiles revealed exceptionally high levels of hydroxytyrosol and rutin. The superior antimicrobial efficacy of these extracts compared with the other extracts suggests that these compounds play a predominant role in the observed bioactivity. In this context, Qanash et al. (2023) reported that rutin exhibits significant antibacterial activity, particularly when its bioavailability is increased. Similarly, Al-Rajhi et al. (2022) demonstrated that higher concentrations of phenolic compounds such as rutin were directly associated with increased antifungal activity. Furthermore, Alsalamah et al. (2023) identified rutin, chlorogenic acid and ferulic acid as key contributors to antimicrobial activity via membrane disruption, as evidenced by ultrastructural analyses.

Although flavonoids such as rutin contribute significantly to antimicrobial activity, OMWExt 2, which is rich in hydroxytyrosol and catechol compounds, exhibited even greater efficacy. These phenolics are characterized by the presence of ortho-dihydroxyl groups, a structural feature that enhances their redox potential and strengthens their interaction with microbial membranes (Schweigert et al., 2001). This configuration promotes membrane destabilization and increases membrane permeability, leading to leakage of intracellular components and impairment of essential cellular functions.

In addition to disrupting the cell membrane, hydroxytyrosol can efficiently penetrate bacterial cells because of its relatively small molecular size and high polarity. Once inside the cell, it interferes with cytoplasmic enzymes, reducing ATP production and altering essential protein functions. Furthermore, hydroxytyrosol has been shown to interact with DNA and inhibit key replication enzymes such as gyrase B, thereby blocking cell replication and microbial proliferation (Ben Hassena et al., 2024). Notably, synthetic analogs of hydroxytyrosol have demonstrated strong antifungal activity, primarily by targeting the plasma membrane, with no evidence of resistance development (Diallinas et al., 2018). Taken together, these findings confirm the role of hydroxytyrosol and catechol phenolic compounds as key contributors to the broad-spectrum antimicrobial activity observed in the present study.

Finally, potential synergistic interactions among phenolic compounds should be considered. The combined presence of hydroxytyrosol, catechol-type phenolic compounds and flavonoids such as rutin may produce additive or synergistic antimicrobial effects. According to Tafesh *et al.*, phenolic compounds tested in combination exhibit greater antimicrobial activity than when evaluated individually (Tafesh et al., 2011).

### 3.6 Statistical analysis

#### 3.6.1 Correlation Pearson test

**Figure 4:**
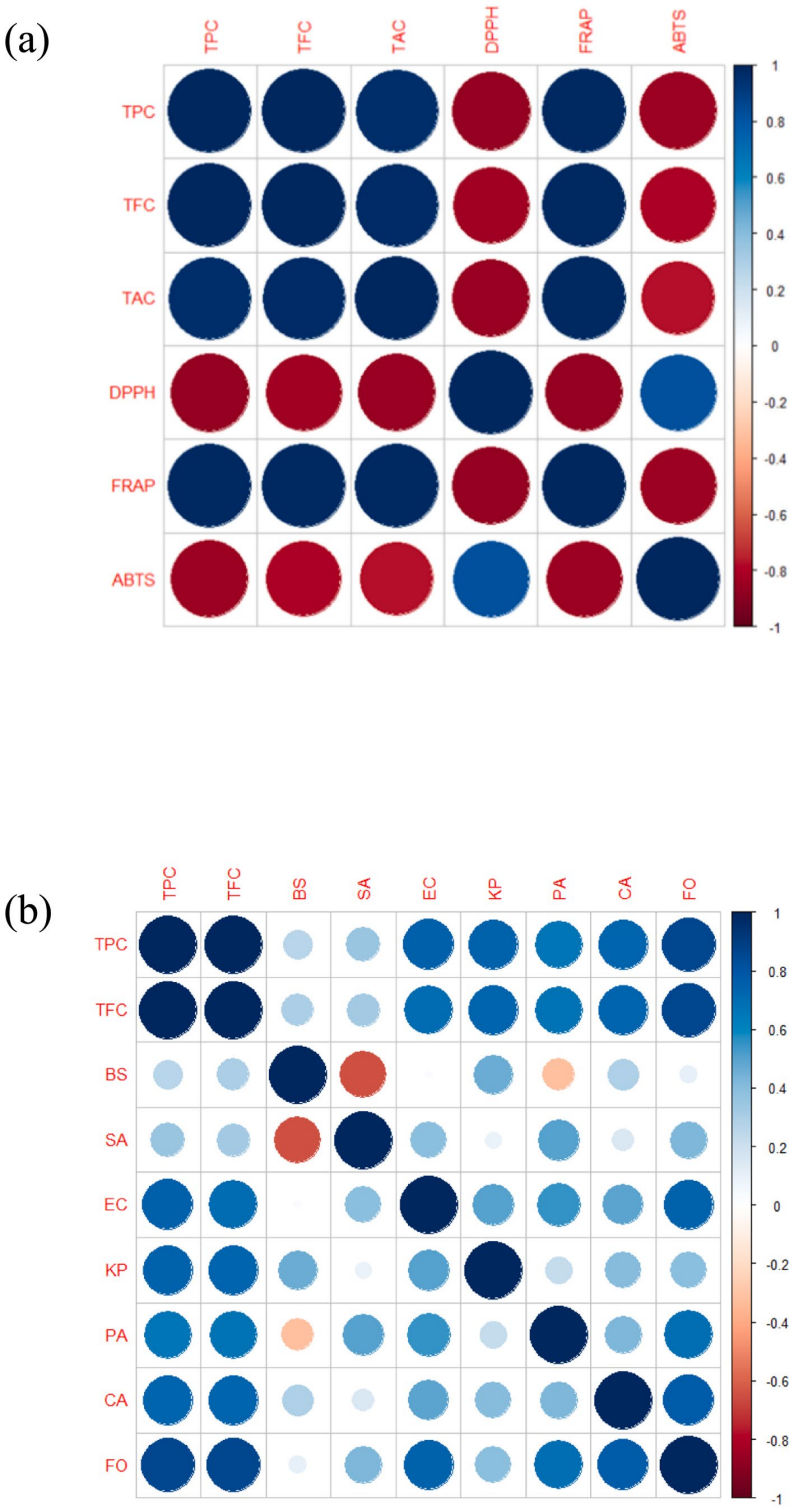
Correlation analysis between total phenolic compound (TPC) and total flavonoid (TFC) concentrations and antioxidant activity **(a)** and antimicrobial activity **(b)**.

Correlation analysis was performed to explore the potential interactions among the parameters studied. This statistical approach is particularly relevant for examining the associations between the contents of bioactive compounds and their biological activities. In this study, correlations between total phenolic content (TPC) and total flavonoid content (TFC), and between antioxidant activity and antimicrobial activity were evaluated via the mean values obtained for the OMWExts. A strong and highly significant positive correlation (r^2^ = 1, r^2^ = 1) was observed between the TPC, TFC, and TAC (Figure 4a). These findings highlighted the central role of phenolic and flavonoid compounds in the antioxidant potential of the extracts. In contrast, the correlations between the TPC, TFC and antioxidant activities measured by the DPPH and ABT assays revealed an inverse correlation, suggesting the involvement of bioactive compounds in the neutralization of free radicals.

Furthermore, a weak correlation was observed between the antimicrobial activity of the extracts and their TPC and TFC contents, indicating that although phenolic compounds increase the antimicrobial potential, their influence is less pronounced than their influence on the antioxidant activity (Figure 4b). These findings are consistent with previous studies that reported strong associations between polyphenolic content and antioxidant activity. However, there is greater variability in the correlation with antimicrobial activity (El Guezzane et al., 2020; Sousa et al., 2008; De Freitas Araújo et al., 2012). Overall, the phenolic richness of the extracts is an important indicator of their bioactive potential, highlighting the relevance of olive by-products as natural sources of high-value compounds.

#### 3.6.2 Principal component analysis

**Figure 5:**
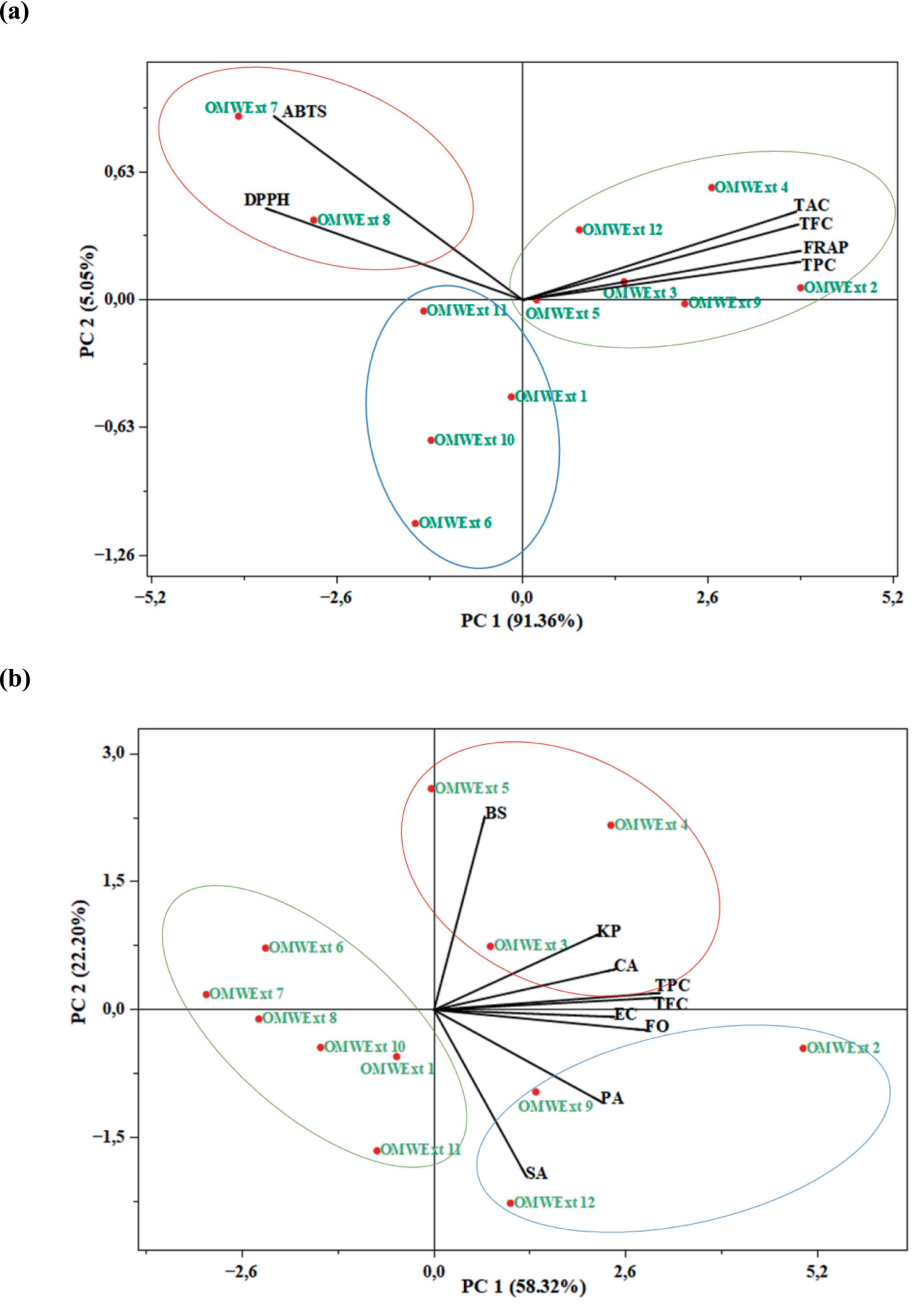
Principal component analysis (PCA): **(a)** Plot showing the relationships between TPC yield, TFC yield, and antioxidant activity (TAC, FRAP, DPPH, ABTS). **(b)** Comparison of the effects of different OMWExts on antimicrobial activity.

The aim of principal component analysis (PCA) is to investigate the variability of all the OMW extracts with respect to their antioxidant and antimicrobial properties, as well as their phenolic compound contents (Figure 5).

Figure 5a shows the positive correlation between the antioxidant activity, total phenolic compound (TPC) content and total flavonoid (TFC) content; the first two components represented 96.41% of the total variance. PC1 (91.36%), which is the principal component, was positively correlated with both the FRAP and TAC assays. These findings suggest that PC1 reflects the overall bioactive compound content and antioxidant potential of the extracts. OMWExts 2, 3, 4, 5, 9, and 12 fall into this category, with strong antioxidant capacity and high phenolic and flavonoid contents.

The second component, PC2 (5.05%), is related primarily to the DPPH and ABTS tests. However, the positive correlation between OMWExt 7 and OMWExt 8 and PC2 indicates decreased scavenging activity because the high values in these two tests indicate lower radical scavenging capacity. The OMWExts 1, 6, 10, and 11 extracts had negative PC1 and PC2 values, indicating a generally weak antioxidant profile with low phenolic content and limited free radical neutralization capacity. This structural variability indicates that geographic origin has a significant effect on chemical composition and antioxidant performance.

Regarding the antimicrobial activity (Figure 5b), the first two components accounted for 80.52% of the total variance, indicating that PCA effectively summarized the associations between the extracts and their biological activities. PC1 (58.32%) was strongly correlated with the level of inhibition observed against the tested bacteria, which was positively impacted by TFC and TPC, indicating that phenolic compound richness affects antimicrobial potential. OMWExts 2, 9, and 12 are in the positive region of the PC1 axis, and have the highest antimicrobial activity, particularly against *S. aureus* and *P. aeruginosa*. The PC2 axis (22.20%) is determined primarily by its activity against *Bacillus subtilis*, whose vector is strongly aligned with the upper part of the factorial plane. OMWExts 3, 4, and 5 are found in this region, indicating that they are active against this Gram-positive strain. In contrast, OMWExts 6, 7, 8, 10, and 11, which are in the negative zones of both axes, exhibit weak antimicrobial potential. Their distance from the most active bacterial vectors indicates minimal, if any, inhibition against all the tested microorganisms.

## 4 Conclusion

This study demonstrated that OMW from different regions of Morocco constitutes a significant by-product of olive oil production characterized by a high organic load and a complex chemical profile. The polyphenolic extracts demonstrated marked variability in chemical composition depending on geographic origin. Seventeen different compounds were identified across the samples, including hydroxytyrosol, which was the predominant compound in 10 of them. All the extracts exhibited strong antioxidant activity, and OMWExt 2 presented the highest performance. Antimicrobial assays revealed that all the extracts were effective against all the bacterial strains tested, while antifungal activity was observed only in extracts with elevated hydroxytyrosol contents.

These findings revealed that the polyphenols present in OMW, particularly hydroxytyrosol, tyrosol, and oleuropein derivatives, significantly contribute to its bioactive potential as valuable sources of bioactive compounds for industrial, pharmaceutical, and agricultural applications. However, the high concentrations of organic matter and phenolic compounds present environmental challenges, accentuating the need for effective management and valorization strategies. The implementation of advanced extraction and purification techniques could enable the recovery of high-value biomolecules, thereby transforming OMW from an environmental pollutant into a sustainable resource. Such approaches support circular economy principles and enhance the overall sustainability of the olive oil industry.

### 4.1 Limitations of the study

We acknowledge that the current study is based on a limited number of olive mill wastewater (OMW) samples collected from five Moroccan regions during one harvest season. As such, the results should be considered preliminary screening data or a proof-of-concept data for the valorization of OMW polyphenols. While the study does not fully capture the variability related to cultivar, mill type, and processing methods, it provides valuable initial insights into the physicochemical, antioxidant, and antimicrobial potential of OMW. Future studies with a larger and more diverse set of samples are planned to expand these findings and validate the observed trends.

## Data Availability

The original contributions presented in the study are included in the article/supplementary material, further inquiries can be directed to the corresponding authors.
